# Exploring the complex dynamics of BMI, age, and physiological indicators in early adolescents

**DOI:** 10.1186/s12887-024-04680-8

**Published:** 2024-04-01

**Authors:** Ning Ding, Suyun Li, Han Zhou, Zhenchuang Tang, Tianlin Gao, Meina Tian, Changqing Liu, Xiaoyan Luo, Hongtong Chen, Lianlong Yu, Yao Chen, Li Yang, Lichao Zhu

**Affiliations:** 1 Department of Pediatrics, Shandong Provincial Hospital Affiliated to Shandong First Medical University, Jinan, Shandong China; 2 Shandong Center for Disease Control and Prevention, Ji’nan, Jinan, Shandong China; 3 Institute of Food and Nutrition Development, Ministry of Agriculture and Rural Affairs, Beijing, China; 4 School of Public Health, Qingdao University, Qingdao, China; 5 Hebei Center for Disease Control and Prevention, Shijiazhuang, Hebei China; 6 Department of cardiothoracic Surgery, Heart Center, Shanghai Children’s Medical Center, Shanghai Jiao Tong University School of Medicine, Shanghai, China; 7 People’s Hospital of Rizhao, Rizhao, Shandong China; 8 Jinan Center for Disease Control and Prevention, Jinan, Shandong China; 9 Department of Pediatric Surgery, Shandong Provincial Hospital Affiliated to Shandong First Medical University, Jinan, Shandong China

**Keywords:** Body Mass Index, Age, Early adolescents, Biochemical index, Blood pressure

## Abstract

**Background and objectives:**

To investigate the relationship between body mass index (BMI) and blood biochemical indicators in early adolescence, and to provide ideas for early prevention of diseases and explore possible disease-related predictors.

**Methods:**

3125 participants aged 10 ∼ 14 years were selected from China from the survey of “China Nutrition and Health Surveillance ( 2016 ∼ 2017 ) “. Employing advanced statistical methods, including generalized linear models, heatmaps, hierarchical clustering, and generalized additive models, the study delved into the associations between BMI and various biochemical indicators.

**Results:**

In early adolescence, indicators including systolic pressure, diastolic pressure, weight, height, BMI, hemoglobin, blood uric acid, serum creatinine, albumin, vitamin A presented increasing trends with the increase of age ( *P* < 0.05 ), whereas LDL-C, vitamin D, and ferritin showed decreasing trends with the increase of age ( *P* < 0.05 ). The increase in hemoglobin and blood uric acid levels with age was more pronounced in males compared to females ( *P* < 0.05 ). BMI was positively correlated with blood glucose, hemoglobin, triglyceride, LDL-C, blood uric acid, serum creatinine, ferritin, transferrin receptor, hs-CRP, total protein, vitamin A ( *P* < 0.05 ). There was a significant BMI × age interaction in the correlation analysis with LDL-C, transferrin receptor, serum creatinine, and hs-CRP ( *P* < 0.05 ). BMI was a risk factor for hypertension, hypertriglyceridemia, low high density lipoprotein cholesterolemia, and metabolic syndrome in all age groups ( OR > 1, *P* < 0.05 ).

**Conclusions:**

High BMI was a risk factor for hypertension, hypertriglyceridemia, low high density lipoprotein cholesterolemia, and MetS in early adolescents. With the focus on energy intake beginning in early adolescence, the maintenance of a healthy weight warrants greater attention.

## Introduction

As a transitional stage between childhood and adulthood [1], adolescence is a very essential period. Adolescents not only differ from adults in physiology, metabolic state, body shape, and organ maturity [2], but also in anthropometric indexes ( e.g. height, weight ) [3], hematology and biochemical indicators. These are associated with a surge of hormones [2], rapid bone growth, eating habits, emotions, and health characteristics during puberty [4].

Height, weight, blood pressure, hematology, and biochemical indicators are routinely used by the medical system to judge the disease or health status of humans [5]. It is essential to study the reference range and variation characteristics of hematology and biochemical indicators in adolescents, which are basic tools for early identification of preventable risk factors and diagnosis of various diseases [1].Also, the appreciation of biochemistry trends in adolescents is essential for understanding the physiology of a growing adolescent and the proper interpretation of laboratory results [6]. BMI is widely used indicator to evaluate nutritional status and is considered the most appropriate method to detect childhood obesity [7]. It has many advantages: cheap, easy to obtain, non-invasive and fast [8]. Studies have shown that BMI has a significant influence on blood biochemistry in adolescence [5]. In contrast, changes in BMI from early to late adolescence are associated with metabolic risk [9]. The psychosocial development can be divided into three stages: Early Adolescence, 10–13 years old; Mid-adolescence, 14–16 years old; Late puberty, 17–18 years old [10]. Early Adolescence is the most active period of growth and development [10], however, previous studies were not systematic and scattered. There is no systematic study on the overall relationship between BMI and Physiological Indicators during the special period of rapid changes in early adolescence. The purpose of our study is to comprehensively understand the relationship between BMI and various indicators in early adolescent children, aiming at a systematic and comprehensive understanding of this physiological period. Therefore, this study aimed to investigate the relationship between changes in blood and biochemical indicators and changes in BMI at this stage of early adolescence, in order to explore relevant predictors for disease prevention and early detection.

## Methods

### Study design

This study is a cross-sectional study conducted in Shandong and Hebei provinces of China from September 2016 to December 2017. The China Nutrition and Health Survey was a nationwide cohort survey. Participants were selected through multi-stage stratified cluster random sampling. Specific sampling methods were as follows: In the first stage, in each monitoring point, 3 towns (streets) were randomly selected by systematic sampling in each monitoring point. In the second stage, two administrative villages (neighborhoods) were randomly selected from each selected township (street) by systematic sampling. In the third stage, in each selected administrative village (neighborhood committee), the households were divided into several villager/resident groups with a scale of not less than 100 households, and each villager/resident group was numbered in turn, and one villager/resident group was selected by simple random sampling method. Phase 4: In each selected villagers/residents group, 60 households were selected by systematic sampling method (according to the population size of the seventh national population census) to carry out the survey, of which the first 30 were dietary survey households and the remaining 30 were non-dietary survey households. Different types of family and family members should complete the corresponding questionnaire, from the “China Nutrition and Health Surveillance ( 2016 ∼ 2017 ) “, and selected 3125 students between the ages of 10 and 14 from Shandong and Hebei province. It has been reviewed by the Ethics Committee of the Chinese Center for Disease Control and Prevention, and the number is 201,614.

The study, which included children 10–14 years old with complete questionnaire information, physical measurement and blood index detection data, excluded children with serious diseases, incomplete questionnaires, physical measurements and data of blood indicators. At the time of our survey, children with fever, obvious infections, and severe illness were not included in the survey, and if high CRP levels were found, it was unknown or unknown to us in advance.

Questionnaire surveys, body examinations, dietary interviews, and laboratory tests were used for collecting information. The body measurement indicators included height, weight, waist circumference, blood pressure. blood and biochemical indicators included serum lipids (TC, TG, HDL-C, LDL-C), fasting blood glucose, high-sensitivity C-reactive protein, ferritin, vitamin A, vitamin D, transferrin receptor, etc. All subjects were collected 6 ml of empty abdominal venous blood, and hemoglobin was detected on the spot. Other indicators are the responsibility of the provincial CDC, according to the state Home project laboratories provide quality control and operational technical requirements to arrange centralized testing.

It has been reviewed by the Ethics Committee of the Chinese Center for Disease Control and Prevention, and the number is 201,614. Subjects gave informed consent and volunteered to participate in this investigation. The informed consent was given by their guardians.

Considering the rapid changes in height and weight during adolescence, we used the quartile for grouping for uniform observation. Participants were classified into quartiles according to BMI levels from the lowest ( the 1st quartile ) to the highest ( the 4th quartile ). Based on the expert consensus on the prevention and treatment of dyslipidemia in Chinese children and adolescents, hypercholesterolemia ( TC ≥ 5.18 mmol/L ), hypertriglyceridemia ( TG ≥ 1.70 mmol/L ), mixed hyperlipidemia ( TC ≥ 5.18 mmol/L and TG ≥ 1.70 mmol/L ) and low high-densitylipoprotein cholesterolemia ( HDL-C ≤ 1.04 mmol/L ) were four types of dyslipidemias [11]. Hypertension was defined by the level of SBP/DBP greater or equal to the 95th percentile ( by age, gender and height ) [12].

The definition of metabolic syndrome (MetS) were based on the criteria of International Diabetes Federation ( IDF ) [13]: Central obesity: Waist circumference ( WC ) ≥ 90th percentile ( P90 ) of the same age and sex is the basic and necessary condition for metabolic syndrome ( MetS ) in adolescents, and it must meet at least two of the following clinical features: (1) fasting blood glucose ≥ 5.6 mmol/L; (2) High triglyceride ( TG ≥ 1.7mmol/L ); (3) Systolic blood pressure ≥ P95 for the same age and sex or diastolic blood pressure ≥ P95 for the same age and sex ( see “China Hypertension Prevention and Treatment Guidelines 2010” designated by the Chinese Hypertension Prevention and Control Guidelines Revision Committee ); (4) Low high-density lipoprotein cholesterol [HDL-C < 1.03mmol/L ( 40 mg/dl )].

Family and personal information were collected by face-to-face interviews and each participant provided overnight fasting blood for measurement of blood biochemical indexes and nutritional status parameters.

Medical examinations were conducted by investigators using standard methods and all measuring instruments complied with the requirements of national metrology certification. The main reference basis of examination methods was the industry standard of the People’s Republic of China-Human Health Monitoring Anthropometric Measurement Method ( WS/T424-2013 ) [14]. Height was measured with a metal column height meter with an accuracy of 0.1 cm and the weight was determined using an electronic scale with an accuracy of 0.1 kg. BMI was computed by weight in kilogram divided by the squared value of height in meters.

Quality control of the survey was performed by national and provincial working groups. Collecting fasting blood samples and undertaking the interviews and questionnaires were responsible by the staff of district and county level uniformly trained by the local Center for Disease Control and Prevention.

### Statistical analysis

In order to compare demographic characteristics and biochemical indicators between different age groups, the χ [2] test for categorical variables and one-way ANOVA for continuous variables were used. Student-Newman-Keuls ( SNK ) tests were used for post hoc comparisons. As large sample data is used in this study, according to the central limit theorem, large sample data tend to be normally distributed, so the measurement data in this study are represented by mean and standard deviation. At the same time, we divided BMI by quartile to explore the relationship between variables. Our study explored the relationship between BMI and biochemical indicators using generalized linear models. The influence of confounders on these relationships was used by stratified and interactions analysis. The visualization of the interaction effect between BMI and age was accomplished utilizing a generalized additive model. Heatmaps and hierarchical clustering were used to explore the relationship between BMI and biochemical indicators. The relationships between various physiological indicators in the study subjects were visualized by employing a network diagram based on correlation coefficients. We calculated ORs ( odds ratios ) and 95% confidence intervals using logistic regression. To analyze dose-response relationships ( using different models ) between BMI, hypertension, and metabolic syndrome ( MetS ), restricted cubic splines ( RCS ) based on logistic regression were used. Graphs were created with the R version 4.1.2, with *P* values at or below 0.05 considered significant.

## Results

A total of 3125 adolescents aged 10 to 14 were surveyed for this study, including 1557 boys and 1568 girls, with a sex ratio of approximately 1:1 for each age group. Table 1 showed the basic demographic characteristics and blood indexes of the study population of different ages. For 10 to 13 years old, each age group took almost 20% of the study population and the 14-year-old group accounted for 13.2%. There was no significant difference in the number of samples in different age groups ( *P* = 0.368 ).

**Table 1:** Sample characteristics according to age ( mean values and standard deviations; numbers and percentages )

s	Total ( *N* = 3125 )		10 years old		11 years old		12 years old		13 years old		14 years old		P values
	Mean	SD	Mean	SD	Mean	SD	Mean	SD	Mean	SD	Mean	SD	
Total ( n, % )	3125 ( 100.0 )		676 ( 21.6 )		679 ( 21.7 )		741 ( 23.7 )		617 ( 19.7 )		412 ( 13.2 )		0.368
Male ( n, % )	1557 ( 49.8 )		319 ( 10.2 )		347 ( 11.1 )		360 ( 11.5 )		322 ( 10.3 )		209 ( 6.7 )		
Female ( n, % )	1568 ( 50.2 )		357 ( 11.4 )		332 ( 10.6 )		381 ( 12.2 )		295 ( 9.4 )		203 ( 6.5 )		
Hypertension ( n, % )	755 (24.2)		147 (4.7)		173 (5.5)		186 (6.0)		126 (4.0)		123 (3.9)		0.004
MetS ( n, % )	76 (2.4)		14 (0.5)		14 (0.5)		18 (0.6)		17 (0.5)		13 (0.4)		0.746
Haemodynamic													
Systolic pressure ( mmHg )	114.9	12.0	111.0	11.5	113.9	11.5	116.6	11.1	116.0	11.7	118.3	13.4	<0.001
Diastolic pressure ( mmHg )	68.4	8.9	67.2	8.6	68.2	8.8	69.0	8.7	68.2	9.2	69.5	9.4	<0.001
Anthropometric													
Weight ( kg )	47.7	13.2	38.5	9.9	44.5	11.5	50.0	12.6	53.1	12.2	55.7	12.7	<0.001
Height ( cm )	154.6	10.5	144.5	7.8	150.6	8.6	157.2	7.8	161.4	8.0	163.1	8.2	<0.001
BMI ( kg/m^2^ )	19.7	4.0	18.2	3.6	19.4	4.0	20.1	4.1	20.3	3.9	20.9	4.0	<0.001
Waist circumference (cm)	67.3	11.5	63.0	10.5	66.2	10.5	68.4	11.3	69.9	13.3	70.6	10.0	<0.001
Biochemistry													
Blood glucose ( mmol/L )	5.2	0.5	5.2	0.5	5.2	0.5	5.2	0.5	5.3	0.6	5.2	0.5	0.362
Hemoglobin ( g/L )	137.7	12.2	134.9	9.7	137.0	10.2	137.9	11.5	139.5	14.3	140.5	15.1	<0.001
Total cholesterol ( mmol/L )	3.9	5.1	4.0	0.7	3.9	0.7	4.2	9.9	3.7	0.7	3.9	3.8	0.504
Triglyceride ( mmol/L )	1.0	1.0	0.9	0.4	1.0	0.4	1.0	0.4	1.0	0.5	1.1	2.4	0.225
HDL-C ( mmol/L )	1.5	3.8	1.5	0.3	1.8	8.1	1.5	0.3	1.4	0.3	1.4	0.3	0.292
LDL-C ( mmol/L )	2.0	0.6	2.1	0.6	2.1	0.6	2.0	0.6	1.9	0.6	1.9	0.6	<0.001
Blood uric acid ( µmol/L )	318.3	79.6	287.9	68.9	305.3	74.5	322.6	78.7	342.0	79.2	345.8	84.7	<0.001
Serum creatinine ( µmol/L )	51.0	9.6	45.5	6.6	47.4	7.3	50.8	8.2	55.6	10.0	59.7	9.8	<0.001
Ferritin ( ng/mL )	54.8	32.5	60.5	30.8	56.6	32.5	51.8	30.5	52.2	34.2	52.1	35.0	<0.001
Transferrin receptor ( mg/L )	3.5	1.2	3.4	0.8	3.5	1.1	3.5	1.1	3.5	1.3	3.5	1.5	0.717
hs-CRP ( mg/L )	1.0	4.6	1.0	2.9	1.3	8.5	0.8	2.4	0.9	2.7	0.8	1.9	0.298
Albumin ( g/L )	49.2	2.9	48.8	2.6	49.2	3.1	48.8	2.8	49.4	3.2	50.0	2.9	<0.001
Total protein ( g/L )	76.0	4.7	75.6	4.6	76.3	4.9	75.6	4.3	76.1	5.0	76.8	4.5	<0.001
Serum Zn ( µg/dL )	91.4	39.5	88.7	19.8	89.0	17.7	96.5	56.5	94.5	54.6	86.3	19.0	0.3028
Vitamin A ( mg/l )	0.4	0.1	0.4	0.1	0.4	0.1	0.4	0.1	0.4	0.1	0.4	0.1	<0.001
Vitamin D ( ng/mL )	15.8	6.0	17.1	5.9	16.5	6.4	15.6	6.1	14.5	5.5	14.8	5.6	<0.001

MetS: metabolic syndrome; SD: standard deviation. The chi-square test was used for the comparison between categorical variables, and generalized linear models were employed for the analysis of continuous variables across groups

**Table 2:** Sample Characteristics according to BMI status ( mean values and standard deviations; numbers and percentages )

	Quartile 1 ( <16.9 kg/m^2^ )		Quartile 2(16.9-18.8 kg/m^2^)		Quartile 3(18.8-21.6 kg/m^2^)		Quartile 4(>21.6 kg/m^2^)		χ^2^/F	P values
	Mean	SD	Mean	SD	mean	SD	mean	SD		
Gender										
Male ( n, % )	371 ( 48.6 )		396 ( 49.0 )		356 ( 46.2 )		427 ( 55.4 )		14.2	0.003
Female ( n, % )	393 ( 51.4 )		412 ( 51.0 )		414 ( 53.8 )		344 ( 44.6 )			
Hypertension ( n, % )	116 (15.2)		160 (19.8)		178 (23.1)		294 (38.1)		125.02	<0.0001
MetS ( n, % )	0		0		0		76 (9.9)		233.0	<0.0001
Age ( n, % )									252.23	<0.0001
10 years old	292 ( 38.2 )		157 ( 19.4 )		111 ( 14.4 )		111 ( 14.4 )			
11 years old	197 ( 25.8 )		170 ( 21.0 )		146 ( 18.9 )		164 ( 21.3 )			
12years old	142 ( 18.6 )		217 ( 26.9 )		181 ( 23.5 )		200 ( 25.9 )			
13years old	95 ( 12.4 )		159 ( 19.7 )		190 ( 24.7 )		169 ( 21.9 )			
14years old	38 ( 5.0 )		105 ( 12.9 )		142 ( 18.4 )		127 ( 16.5 )			
Blood glucose ( mmol/L )	5.2	0.5	5.2	0.6	5.3	0.5	5.3	0.5	6.2	<0.001
Hemoglobin ( g/L )	135.6	10.2	138.1	12.0	138.4	13.0	138.8	13.0	10.6	<0.001
Total cholesterol ( mmol/L )	3.9	0.6	3.9	2.7	4.1	9.8	3.9	0.7	0.5	0.7018
Triglyceride ( mmol/L )	0.9	0.3	0.9	0.4	0.9	0.4	1.2	1.8	17.8	<0.001
HDL-C ( mmol/L )	1.6	0.3	1.5	0.3	1.4	0.3	1.6	7.6	0.3	0.8287
LDL-C ( mmol/L )	2.0	0.6	1.9	0.6	2.0	0.6	2.1	0.6	18.8	<0.001
Blood uric acid ( µmol/L )	282.5	62.8	305.7	72.9	324.2	74.8	360.9	85.0	152.6	<0.001
Serum creatinine ( µmol/L )	47.6	8.0	50.8	9.3	53.0	10.3	52.8	9.8	54.4	<0.001
Ferritin ( ng/mL )	56.8	29.3	53.0	29.7	51.6	32.8	58.0	37.5	6.7	0.001
Transferrin receptor ( mg/L )	3.3	0.9	3.3	1.0	3.5	1.3	3.8	1.3	11.3	<0.001
hs-CRP ( mg/L )	0.7	2.1	0.7	2.6	0.8	2.1	1.8	8.2	11.3	<0.001
Albumin ( g/L )	48.9	2.7	49.1	2.7	49.3	3.1	49.4	3.2	5.6	<0.001
Total protein ( g/L )	75.1	4.4	75.6	4.8	76.2	4.8	77.2	4.6	28.6	<0.001
Serum Zn ( µg/dL )	89.78	29.11	92.61	47.74	91.67	34.95	91.63	43.18	0.50	0.4814
Vitamin A ( mg/l )	0.4	0.1	0.4	0.1	0.4	0.1	0.4	0.1	43.4	<0.001
Vitamin D ( ng/mL )	16.3	5.8	15.5	6.1	15.4	5.9	16.0	6.1	3.7	0.0111

MetS: metabolic syndrome; The chi-square test was used for the comparison between categorical variables, and generalized linear models were employed for the analysis of continuous variables across groups.

**Fig. 1:**
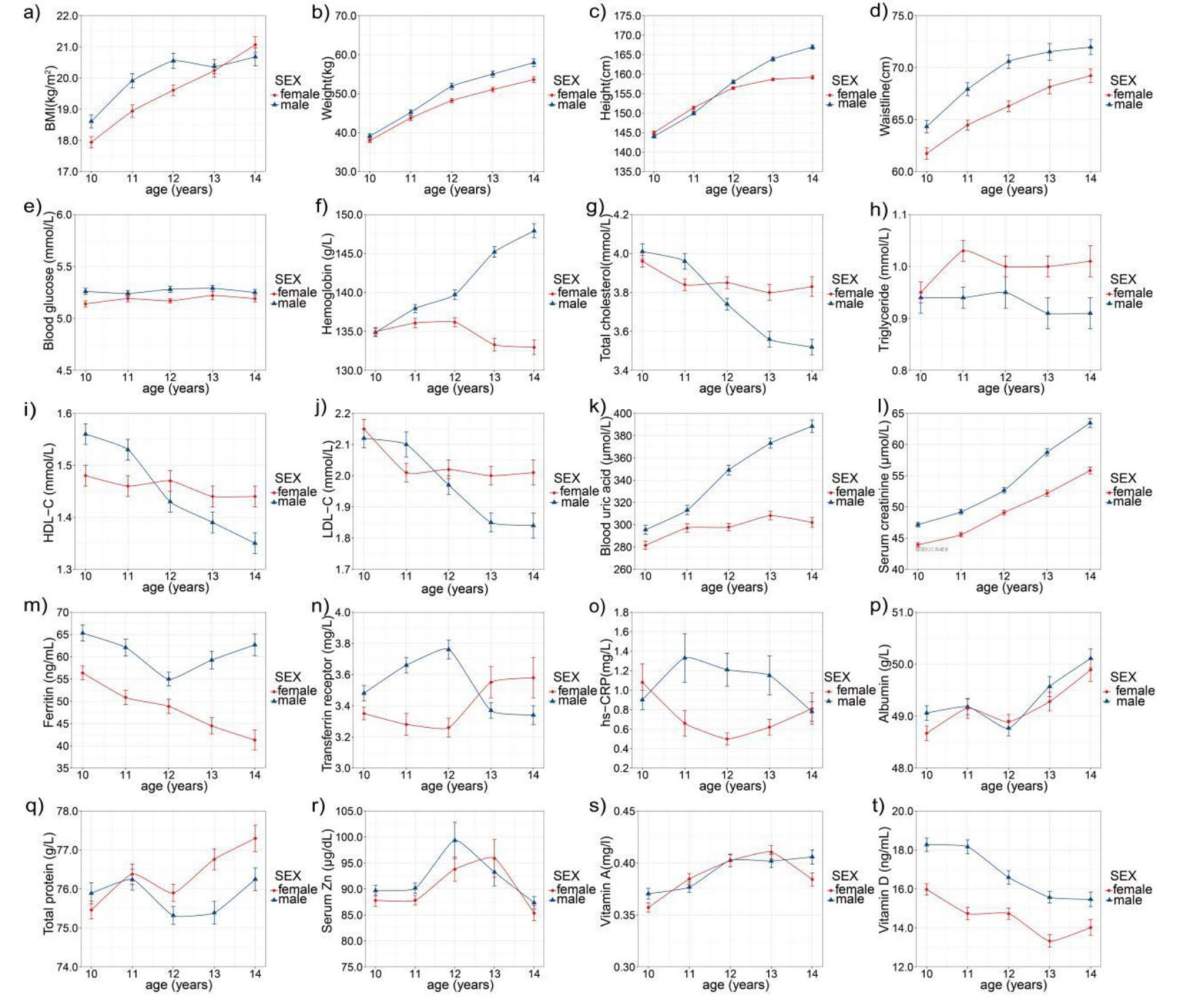
Changes in physiological indicators with age in Early Adolescent children by gender. a ) BMI with age. b ) Weight with age. c ) Height with age.d ) Waistline with age. e ) Blood glucose with age. f ) Hemoglobin with age. g ) Total cholesterol with age. h ) Triglyceride with age. i ) HDL-C with age. j ) LDL-C with age. k ) Blood uric acid with age. l ) Serum creatinine with age. m ) Ferritin with age. n ) Transferrin receptor with age. o ) hs-CRP with age. p ) Albumin with age. q ) Total protein with age. r ) Serum Zn with age. s ) Vitamin A with age. t ) Vitamin D with age. Data are mean values ± standard errors

Table 1 showed that there was no statistically significant difference in blood glucose, total cholesterol, triglyceride, HDL-C, transferrin receptor, and hs-CRP distribution among different age subgroups ( *P* > 0.05 ). However, there were statistically significant differences in systolic pressure, diastolic pressure, weight, height, BMI, hemoglobin, blood uric acid, serum creatinine, albumin, total protein, and vitamin A distribution among various age subgroups. On the whole, the indicators including systolic pressure, diastolic pressure, weight, height, BMI, hemoglobin, blood uric acid, serum creatinine, albumin, vitamin A presented increasing trends with the increase of age, whereas LDL-C, vitamin D, and ferritin showed decreasing trends with the increase of age ( *P* < 0.001 ).

Indicators such as TC, HDL-C, and LDL-C showed intersecting trends at certain age points. Moreover, the increase in hemoglobin and blood uric acid levels with age was more pronounced in males compared to females. Figure 1 objectively reflects the alterations in blood indicators during early puberty.

As shown in Table 2, there were statistically substantial differences in gender and age distribution among different BMI groups ( *P* < 0.01 ). Meanwhile, as observed from Fig. 1, the change trend of BMI in different genders with ages indicated that the value of BMI generally rose with age. Subjects with a lower level of BMI status had significantly lower blood glucose, hemoglobin, triglyceride, blood uric acid, serum creatinine, transferrin, transferrin receptor, hs-CRP, albumin, total protein, and vitamin A ( *P* < 0.01 ). However, there were no significant differences in total cholesterol, HDL-C, and serum zinc among various BMI groups ( *P* > 0.05 ).

**Table 3:** Multi-variable associations of BMI with Blood Biomarkers in Chinese Early Adolescents

		Model 1			Model 2		P-interaction BMI* age
Blood biochemical indexes	coefficient	95%CI	*P*	coefficient	95%CI	*P*	
Blood glucose ( mmol/L )	0.009	( 0.004,0.013 )	<0.001	0.088	( 0.004,0.013 )	<0.001	0.899
Hemoglobin ( g/L )	0.208	( 0.105,0.312 )	<0.001	0.012	( 0.016,0.227 )	0.023	0.557
Total cholesterol( mmol/L )	0.009	( -0.035,0.053 )	0.685	0.013	( -0.032,0.058 )	0.573	0.987
Triglyceride ( mmol/L )	0.028	( 0.019,0.036 )	<0.001	0.028	( 0.019,0.036 )	<0.001	0.609
HDL-C ( mmol/L )	0.014	( -0.048,0.018 )	0.377	0.011	( -0.045,0.023 )	0.525	0.743
LDL-C ( mmol/L )	0.018	( 0.013,0.023 )	<0.001	0.023	( 0.018,0.028 )	<0.001	0.021
Blood uric acid ( µmol/L )	7.180	( 6.558,7.803 )	<0.001	6.403	( 5.781,7.025 )	<0.001	0.746
Serum creatinine ( µmol/L )	0.396	( 0.315,0.477 )	<0.001	0.159	( 0.087,0.231 )	<0.001	0.012
Ferritin ( ng/mL )	0.288	( 0.006,0.569 )	0.045	0.472	( 0.187,0.758 )	0.001	0.235
Transferrin receptor ( mg/L )	0.045	( 0.035,0.055 )	<0.001	0.047	( 0.036,0.057 )	<0.001	0.014
hs-CRP( mg/L )	0.135	( 0.096,0.176 )	<0.001	0.147	( 0.107,0.188 )	<0.001	0.002
Albumin ( g/L )	0.035	( 0.009,0.061 )	0.0080	0.019	( -0.006,0.046 )	0.1360	0.435
Total protein ( g/L )	0.178	( 0.137,0.218 )	<0.001	0.173	( 0.132,0.215 )	<0.001	0.539
Serum Zn ( µg/dL )	0.080	-0.267,0.428 )	0.6840	0.045	( -0.310,0.410 )	0.8023	0.433
Vitamin A( mg/l )	0.005	( 0.004,0.006 )	<0.001	0.005	( 0.004,0.005 )	<0.001	0.162
Vitamin D ( ng/mL )	0.032	( -0.084,0.019 )	0.228	0.018	( -0.034,0.070 )	0.158	0.135

Generalized linear models were utilized for model analysis ( All the coefficients mentioned in the table refer to Beta coefficients )

Model1: adjusted for gender ( male/female ); Model2: adjusted for gender ( male/female ) and age.

After multi-variable adjustment, associations between BMI and blood biochemical indexes were presented in Table 3, in which model 1 was adjusted for gender, while model 2 was for age and gender. BMI was positively correlated with blood glucose, hemoglobin, triglyceride, LDL-C, blood uric acid, serum creatinine, ferritin, transferrin receptor, hs-CRP, total protein, vitamin A in all models. There was a significant BMI × age interaction in the correlation analysis with LDL-C, transferrin receptor, serum creatinine, and hs-CRP ( *P* < 0.05 ).

**Fig. 2:**
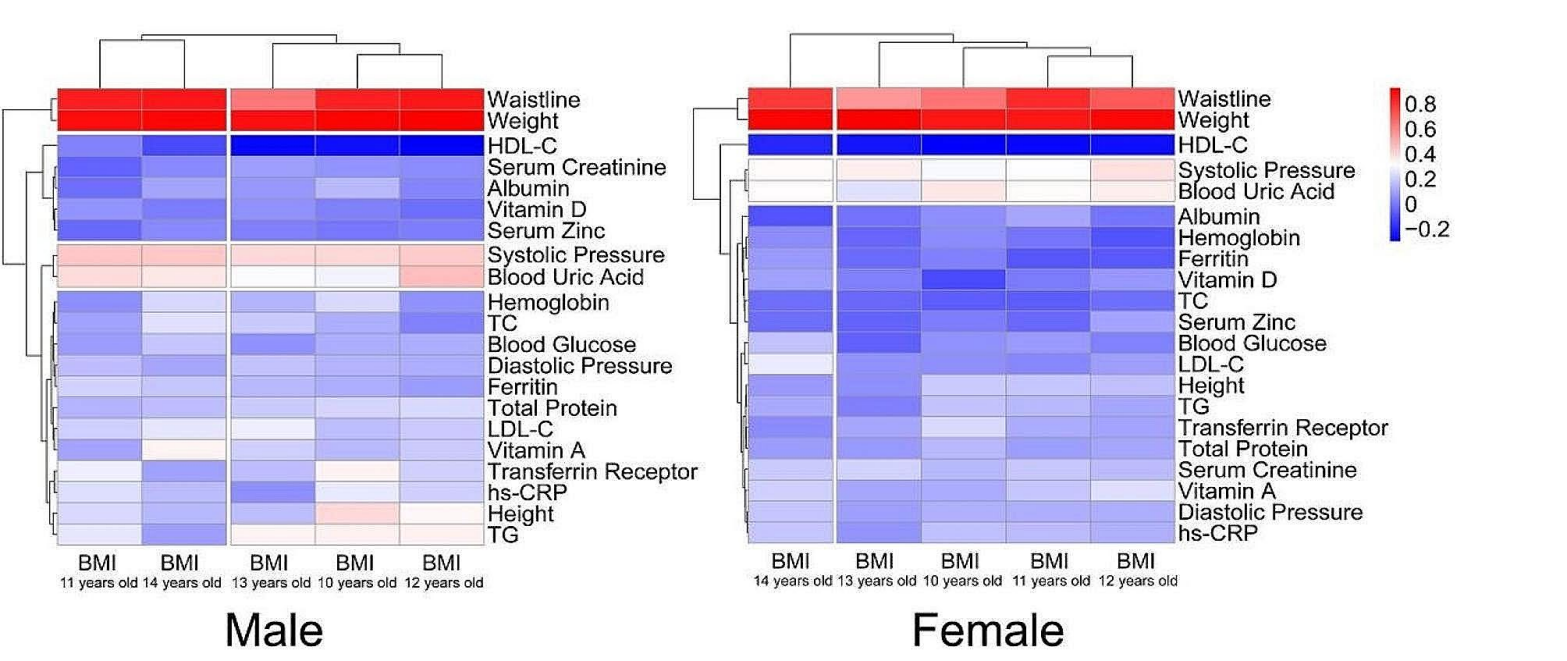
Clustering and correlation heat map between BMI according to age and physiological indicators in preadolescent children by gender

Heatmaps and hierarchical clustering were shown in Fig. 2. According to the correlation between each blood indicator and BMI, there are differences in the clustering of biomarkers between males and females. However, both systolic pressure and blood uric acid are categorized into the same cluster in both male and female populations.

As shown in Table 4, BMI was a risk factor for hypertension, hypertriglyceridemia, low high density lipoprotein cholesterolemia, and metabolic syndrome in all age groups ( OR > 1, *P* < 0.05 ). Additionally, BMI was a risk factor for mixed hyperlipidemia in the 11 and 14 years old group. Furthermore, BMI was a risk factor for hypercholesterolemia in 14 years old group ( OR > 1, *P* < 0.05 ).

**Fig. 3:**
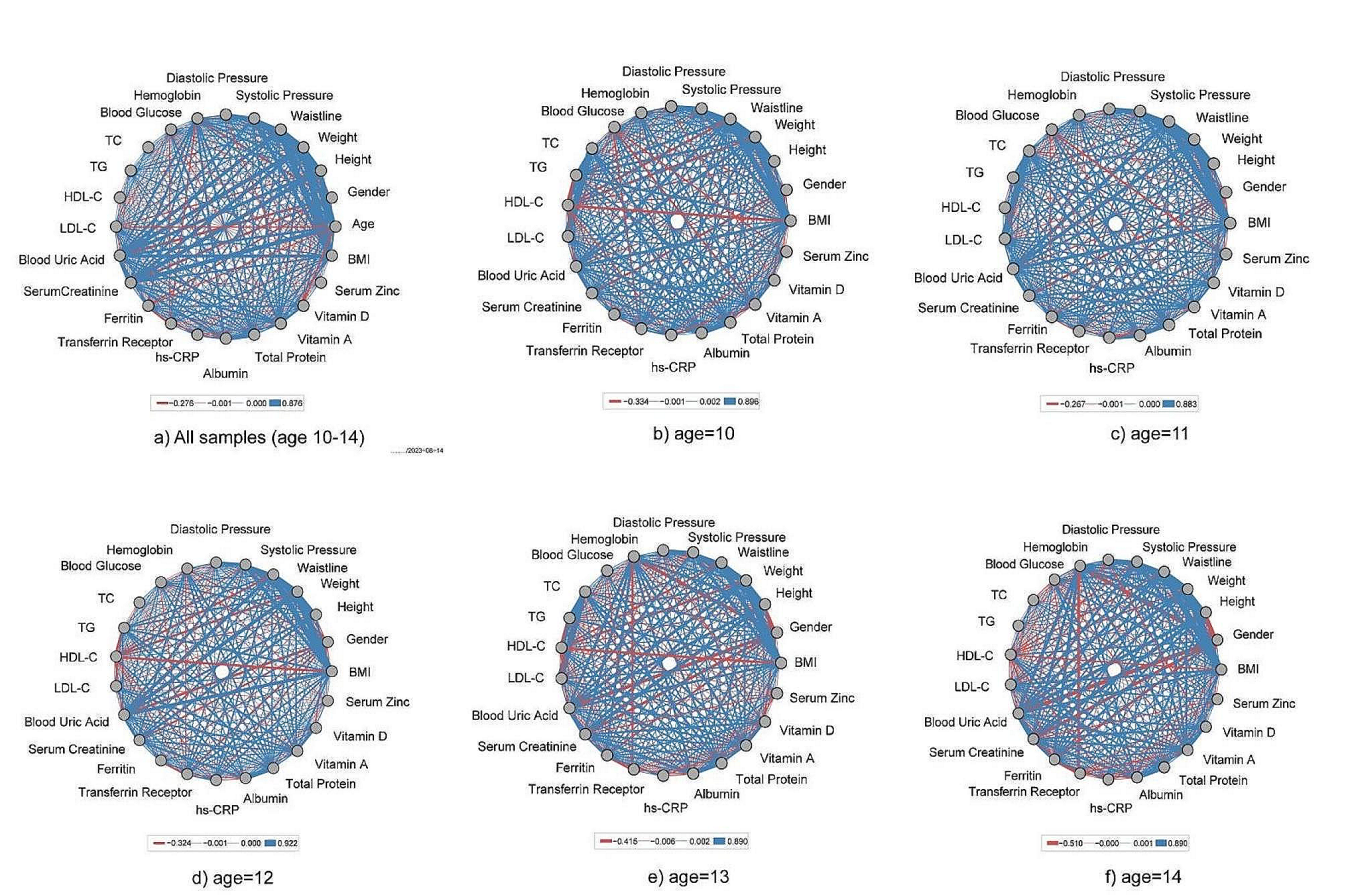
Pearson correlation coefficient network of physiological indicators of preadolescent children by age

Figure 3 illustrates a correlation network graph displaying various physiological indicators among children and adolescents aged 10 to 14, with each year of age being represented. The thickness of the lines denotes the strength of the correlations, with red indicating negative correlations and blue indicating positive correlations. The graph reveals that as age progresses, intricate alterations manifest in the correlations among physiological indicators in pre-adolescent children and adolescents.

**Table 4:** Logistic regression analysis of relationship between BMI and risk factors in different ages

	10 years old			11 years old			12 years old			13 years old			14 years old		
	OR	95% CI	P	OR	95% CI	P	OR	95% CI	P	OR	95% CI	P	OR	95% CI	P
Hypertension	1.08	( 1.03,1.13 )	0.002	1.11	( 1.07,1.16 )	<0.001	1.13	( 1.09,1.18 )	<0.001	1.178	( 1.121,1.24 )	<0.001	1.17	( 1.11.1.24 )	<0.001
Hypercholesterolemia	1.07	( 0.98,1.17 )	0.122	1.04	( 0.96,1.13 )	0.365	1.09	( 1.00,1.20 )	0.063	1.049	( 0.93,1.18 )	0.472	1.15	( 1.02,1.30 )	0.018
Hypertriglyceridemia	1.18	( 1.10,1.27 )	<0.001	1.14	( 1.061,1.21 )	<0.001	1.15	( 1.08,1.22 )	<0.001	1.097	( 1.03,1.18 )	0.008	1.20	( 1.11,1.30 )	<0.001
Mixed hyperlipidemia	1.15	( 0.95,1.39 )	0.167	1.26	( 1.01,1.58 )	0.044	1.11	( 0.90,1.38 )	0.318	1.088	( 0.88,1.35 )	0.435	1.38	( 1.12,1.71 )	0.003
Low high density lipoprotein cholesterolemia	1.15	( 1.08,1.23 )	<0.001	1.11	( 1.04,1.18 )	0.001	1.13	( 1.07,1.19 )	<0.001	1.124	( 1.06,1.19 )	<0.001	1.10	( 1.02,1.18 )	0.011
Metabolic syndrome	1.52	( 1.32,1.76 )	<0.001	1.46	( 1.28,1.68 )	<0.001	1.44	( 1.30,1.61 )	<0.001	1.892	( 1.51,2.37 )	<0.001	1.64	( 1.36,1.96 )	<0.001

Logistic regression was used for model analysis

**Fig. 4:**
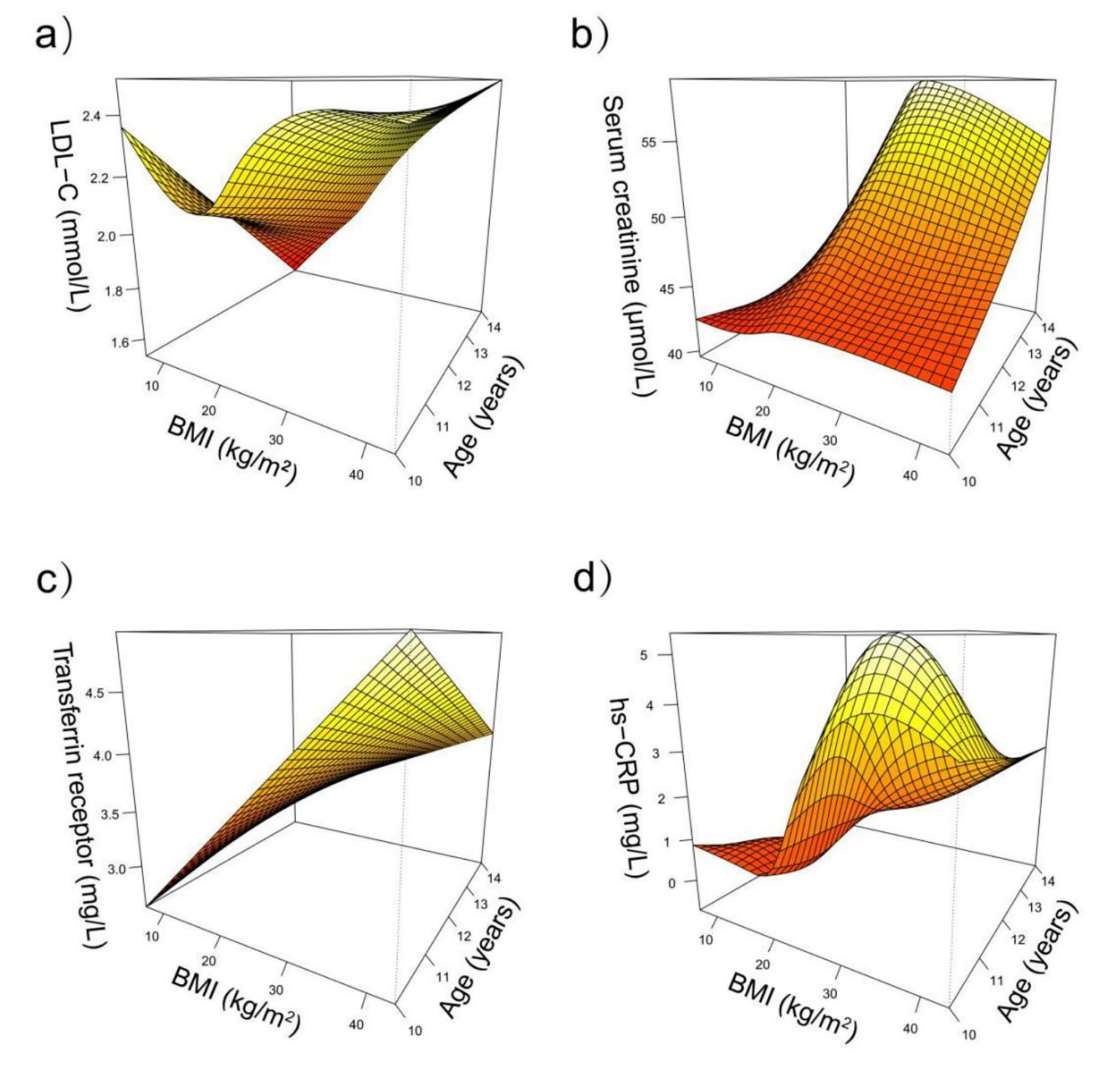
Estimated effects for the tensor product smooth interaction BMI×Age in a semiparametric model. a ) Effects of BMI and age interaction on LDL-C. b ) Effects of BMI and age interaction on Serum creatinine. c ) Effects of BMI and age interaction on Transferrin receptor. d ) Effects of BMI and age interaction on hs-CRP.

Figure 4 visualizes the interaction effect of BMI and age in the correlation analysis of low-density lipoprotein cholesterol ( LDL-C ), transferrin receptor, serum creatinine, and high-sensitivity C-reactive protein ( hs-CRP ). The relationship between LDL-C and BMI shows a U-shaped pattern in the 10-year-old group, which gradually transitions to a linear positive correlation as age increases until 14 years old ( Fig. 4a ). The relationship between serum creatinine and BMI exhibits an inverted U-shaped pattern in the age range of 10 ∼ 14 years old, with this relationship becoming gradually more pronounced as age increases. Additionally, serum creatinine levels gradually increase with age ( Fig. 4b ). The positive correlation between transferrin receptor and BMI weakens as age increases ( Fig. 4c ). The relationship between hs-CRP and BMI becomes relatively stable as BMI increases. When BMI reaches 15 kg/m², Hs-CRP levels start to rapidly increase. However, when BMI reaches 28 kg/m^2^, hs-CRP begins to decline. It is important to note that the intensity of this relationship between hs-CRP and BMI varies with age, with the steepest slope observed in the 11- and 12-year-old populations ( Fig. 4d ).

**Fig. 5:**
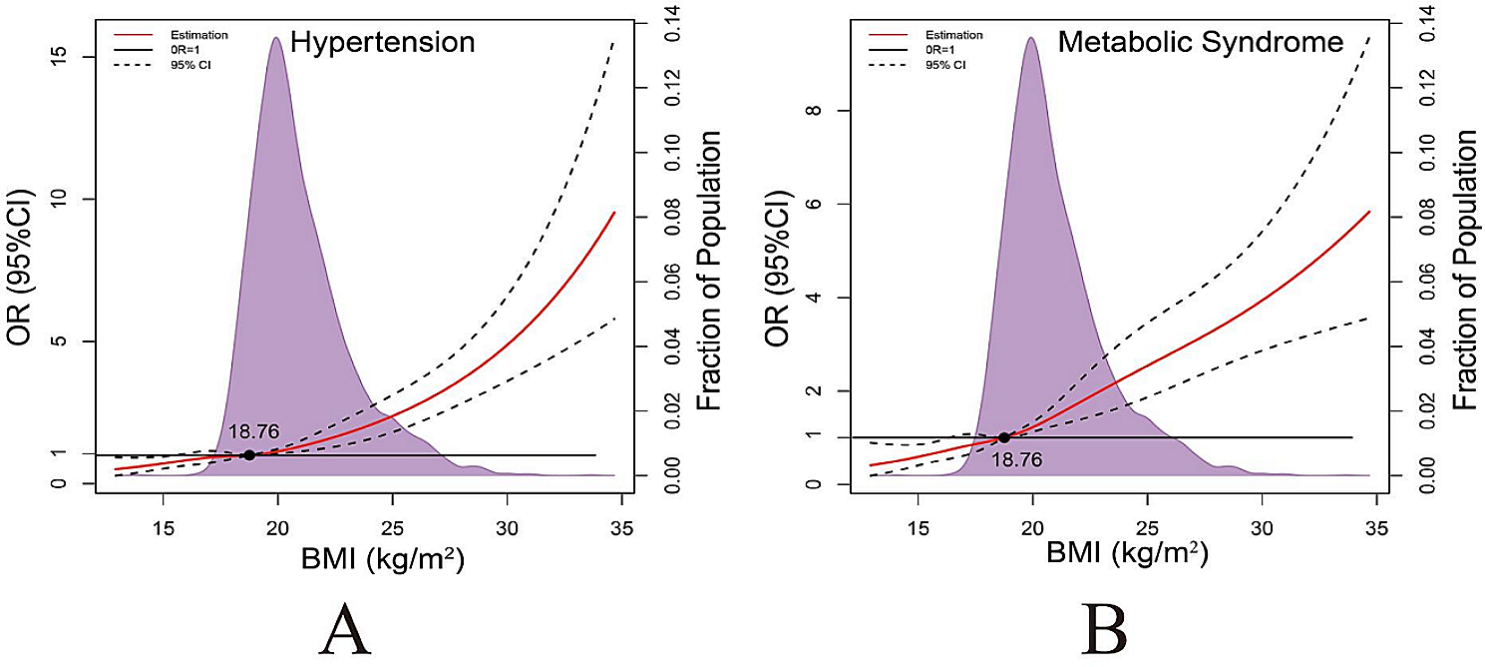
Representation of restricted cubic spline logistic regression models for BMI and risk of hypertension and metabolic syndrome. Red solid line shows OR as a function of BMI adjusted for gender

Figure 5 incorporated BMI as a continuous variable into the RCS model to objectively and accurately display the dose relationship between BMI, hypertension and MetS. According to the RCS curve drawn in the present study, it indicated that the associations of the ORs of hypertention and MetS with the increase in BMI.

## Discussion

This research investigated the blood biochemical parameters in early adolescents, considering their BMI and age. Significant variations were observed in hemoglobin, blood uric acid, serum creatinine, albumin, total protein, LDL-C, ferritin, vitamin A, and vitamin D across different age groups. Additionally, a positive correlation was found between blood glucose, hemoglobin, triglycerides, LDL-C, blood uric acid, serum creatinine, ferritin, transferrin receptor, hs-CRP, albumin, total protein, and vitamin A with BMI. This association remained significant even after controlling for age and gender. Age has a significant effect on the relationship between BMI and LDL-C, transferrin receptor, serum creatinine, and hs-CRP in a prepubertal population. With the increase of BMI in early adolescence, OR of various diseases increased. These findings provided a scientific foundation for the control and prevention of common chronic diseases in early adolescence.

As expected, this study observed anthropometric indexes and biomarkers rapidly changed with age in early adolescence. The most essential feature of early adolescence lies in that the secretion of various hormones increases sharply, and there are three adjacent age groups ( boys aged 12 ∼ 14 and girls aged 10 ∼ 12 ) with the fastest growth in height ( growth spurt period ) [15].During this period, the increasing rate of weight was higher than that of height, and BMI increased with age [16–18], which was in line with the results of our study. As shown in Table 1, there was no difference in TC, TG, and HDL-C among different age groups. LDL-C decreased with age, and identical conclusions were drawn in a cohort study conducted in Denmark [19]. The reason why LDL-C decreases with age has not been fully clarified, and it might be related to height [20, 21]. Meanwhile, several studies indicated that short stature was associated with increased LDL-C levels and coronary artery diseases of adults, identifying a negative correlation between LDL-C and height which might be because growth hormones ( GH ) play a certain role in American and South Korea population [22, 23]. Clinically, LDL-C of patients who undergo GH replacement therapy to increase height growth velocity remarkably decreases with the increased height, implying that the dynamic GH either directly or indirectly influences LDL-C level [24]. Our results indicate that albumin and total protein level increases after 12 years old, which is partially consistent with the results of a study in China [25]. This may be due to the large amount of nutrients needed to build up in the body during puberty. A similar study that investigated biochemical trends among 0 to 18-year-old groups drew the same conclusion that albumin levels in children and adolescents varied at different stages of growth and have not yet reached the adult levels [6]. Meanwhile, the Canadian Laboratory Initiative on Pediatric Reference Intervals ( CALIPER ) project demonstrated that there were age differences in the total serum protein and albumin in children and adolescents [26, 27]. The relationship between age and biochemical indicators in this study is a guide for young children and adolescents early in adolescence.

Biomarkers were linked to BMI in early adolescence, but age should be considered as a factor. As shown in Table 3, the relationship between BMI and LDL-C was affected by age through interaction analysis. The relationship between BMI and LDL-C was influenced by age in children and adolescents aged 2–17 years [28]. A 5-year follow-up study suggested that BMI and LDL-C had different effects on children of different grades in Japan [29]. Similarly, the relationship between BMI and renal function was also affected by age [30]. There was a cohort study showing a U-shaped association between BMI and renal function that was more prominent with increasing age [31]. Our study found that age affected the association between BMI and serum creatinine in early adolescence, which was also in agreement with a survey in China’s Xinjiang. Among children aged 6 ∼ 17, serum creatinine significantly correlated with BMI in children aged over 10, and the correlation coefficient increased with age [32]. The mechanism of this interaction remains unclear, but it was related to the continuous development of the kidney in the early youth [33], and growth hormone can promote the growth of the kidney and the change of renal function [34]. Boys experienced muscle growth and changes in BMI as they grew. Studies have found that transferrin receptors increased with BMI [35], and obese individuals had higher levels of soluble transferrin receptors compared to overweight and normal-weight individuals [36]. Obesity is associated with chronic inflammation due to the production of pro-inflammatory molecules by adipose tissue. BMI is linked to serum inflammatory markers like hs-CRP, which is produced in the liver and influenced by cytokines and hormones [37]. Aging and changes in sex hormones and leptin secretion can impact hs-CRP synthesis [15, 38, 39].

According this study, as BMI increases in early adolescence, OR of various diseases also increase, such as hypertension. In China, the prevalence of hypertension varies from 5 to 30% from children to adults [40]. During the observed period, there was a notable correlation between an elevation in BMI/WC and a heightened occurrence of hypertension [41]. Findings from a retrospective cohort analysis involving individuals from three U.S. healthcare networks indicated that adolescents aged 12 to 17 years who transitioned to or maintained obesity status exhibited a threefold greater likelihood of developing hypertension in comparison to those who maintained a normal weight over a median follow-up duration of 3.1 years [42]. Epidemiological evidence suggests that atherosclerosis initiation commences in childhood, with dyslipidemia identified as the primary risk factor for cardiovascular disease, typically originating in childhood as well [43]. Several studies have shown a significant correlation between childhood and adult lipid levels [44]. The results of one study showed a steady increase in mean TC levels with increasing BMI, and this increase was statistically significant. The individual increases for boys and girls were also statistically significant [45]. According to Friedland et al., obese children with a BMI > 85% had significantly higher mean serum cholesterol and TG levels ( *P* < 0.05 ) [46]. In the study by Zhang et al., Chinese obese children had lower levels of HDL-C and higher levels of TG, LDL-C and significantly higher prevalence of TG and LDL-C dyslipidemia [47]. According to another study, the prevalence of LDL-c dyslipidemia increased with BMI in all participants ( *P* = 0.007 ). Although this was also true in individual girls ( *P* = 0.018 ), the prevalence of LDL-c dyslipidemia was not statistically significantly different from the increase in individual BMI in boys ( *P* = 0.272 ) [45]. According to other literature, with increasing BMI, mean TG increased for all participants, boys and girls. In addition, the prevalence of hypertriglyceridemia increased in all participants ( both boys and girls ) with increasing BMI. In summary, overall dyslipidemia increased with increasing BMI [45]. According to previous studies, there was closely correlation between metabolic syndrome and BMI rather than other measures of body composition [48].

Our results clearly demonstrate the divergent trajectories of these indicators between males and females during early puberty. These indicating distinct physiological changes occurring in males and females during this critical developmental stage. Furthermore, we also identified an interaction between BMI and age in the indicators of LDL-C, serum creatinine, Transferrin receptor, and hs-CRP. Overall, our findings highlight the importance of considering gender and age specific variations when interpreting blood indicator changes during early puberty. The observed differences in the trajectories of these physiological markers provide valuable insights into the diverse physiological processes occurring during this crucial period of development.

There are several limitations in our research. For one thing, the data were based on cross-sectional study,which was not sufficient for a causal assessment of the relationship between the variables in the study. For another thing,we only observed early adolescents ( aged 10 ∼ 14 )which might not reflect the relationship between BMI and blood lipid level and dyslipidemia in the whole adolescents. These questions will be refined in future research. Since this study is based on surveillance data, although there are sample size calculations based on large populations, there is still a lack of previous sample size calculations for the purpose of this study. This study has more exploratory purpose and significance. Due to the initial design, the influences of menstrual status, sex hormone levels, exercise and dietary intake data were not fully considered. We will refine this part in future research.

## Conclusions

In early adolescence, indicators including systolic pressure, diastolic pressure, weight, height, BMI, hemoglobin, blood uric acid, serum creatinine, albumin, vitamin A presented increasing trends as age increased, whereas LDL-C, vitamin D, and ferritin showed decreasing trends.The increase in hemoglobin and blood uric acid levels with age was more pronounced in males compared to females. BMI was positively correlated with blood glucose, hemoglobin, triglyceride, LDL-C, blood uric acid, serum creatinine, ferritin, transferrin receptor, hs-CRP, total protein, vitamin A. There was a significant BMI×age interaction in the correlation analysis with LDL-C, transferrin receptor, serum creatinine, and hs-CRP. As age increasing, complex changes occur in the correlations between BMI and physiological indicators in pre-adolescent children and adolescents.With the increase in BMI, the effect of BMI switched from protective to pathogenic for hypertention and MetS.

## Data Availability

The data in this study are owned by the Chinese Center for Disease Control and Prevention, and data sharing is not supported at this time, but data supporting the results of this study are available from the corresponding author on reasonable request.
